# High-density EEG study in patients with schizophrenia treated with cariprazine

**DOI:** 10.1093/ijnp/pyag008

**Published:** 2026-03-09

**Authors:** Pál Czobor, Brigitta Kakuszi, Máté Fullajtár, István Bitter

**Affiliations:** Department of Psychiatry and Psychotherapy, Semmelweis University, Budapest, Hungary; Department of Psychiatry and Psychotherapy, Semmelweis University, Budapest, Hungary; Department of Psychiatry and Psychotherapy, Semmelweis University, Budapest, Hungary; Department of Psychiatry and Psychotherapy, Semmelweis University, Budapest, Hungary

**Keywords:** schizophrenia, cariprazine, resting-state EEG, EEG spectral power, gamma activity

## Abstract

**Background:**

Schizophrenia is marked by persistent negative and cognitive symptoms that remain difficult to treat. Cariprazine, a dopamine D3/D2 partial agonist, has demonstrated efficacy in these domains, making it essential to clarify its neurophysiological mechanisms both for future drug discovery and clinical application. Resting-state EEG spectral analysis provides a non-invasive and translational tool for monitoring neurophysiological changes related to treatment. However, data on EEG spectral effects in human patients treated with cariprazine are currently lacking.

**Methods:**

In this open-label investigation, 27 patients with schizophrenia underwent high-density resting-state EEG before and during cariprazine treatment. Power spectral analyses were conducted across frequency bands and cortical regions, with ROI analyses targeting gamma-activity in DLPFC, IFG, and TPJ. Symptom changes were measured using the Positive and Negative Syndrome Scale.

**Results:**

Cariprazine treatment led to symptom improvement, particularly in negative and general domains. EEG analyses showed increased spectral power, most prominently in the theta and alpha bands, mirroring findings from the only available in vivo preclinical study, which also reported theta and alpha elevations in rodents. Symptom reduction was specifically associated with increased theta and decreased delta power. Moreover, baseline gamma-activity—especially in the IFG and DLPFC—predicted treatment response, with Receiver Operating Characteristic analyses yielding AUC values of 0.72-0.92 and cross-validated classification accuracies exceeding 70%.

**Conclusions:**

Together, our results demonstrate that resting-state EEG spectral measures reflect neurophysiological correlates of cariprazine response. Furthermore, the findings reinforce the cross-species translatability of spectral measures and support EEG biomarkers as promising translational tools for predicting and monitoring treatment outcomes in schizophrenia.

## Graphical Abstract

**Graphical Abstract:**
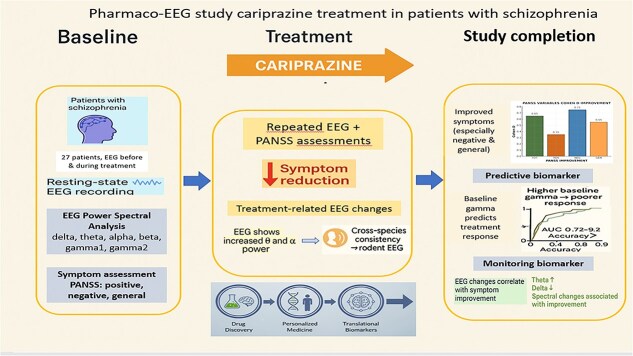

## Teaser

- Resting-state EEG captured neurophysiological changes during cariprazine treatment.
- Treatment response was linked to changes in resting-EEG spectral measures, including increased theta and decreased delta power.
- Baseline gamma activity predicted treatment response with high accuracy.

**Significance statement**

Schizophrenia is a severe mental illness where negative symptoms and cognitive problems often persist despite treatment. Cariprazine, a medication targeting dopamine D3/D2 receptors, has shown promise for these difficult symptoms, but its neurobiological effects in patients require further clarification. In this study, we used high-density resting-state EEG, a safe and non-invasive method, to measure brain activity in patients with schizophrenia before and during cariprazine treatment. We found that cariprazine improved symptoms, especially negative ones, and was linked to specific brain activity patterns: increased EEG theta and alpha power and changes in delta power. Notably, baseline gamma activity predicted who would respond to treatment, with strong accuracy. These findings highlight EEG’s potential as a practical tool for guiding clinical trials for patient selection and developing personalized treatment strategies.

## Background

Schizophrenia is a severe and disabling psychiatric disorder affecting approximately 1% of the population worldwide. It is characterized by profound disturbances in perception, thought, emotion, and behavior, manifesting as positive symptoms (eg, delusions, hallucinations, disorganized thinking), negative symptoms (eg, affective flattening, alogia, avolition), and cognitive impairments. These symptom domains contribute to long-term functional disability, with negative symptoms and cognitive deficits being especially associated with poor response to treatment.

Second-generation antipsychotics are considered first-line treatments due to their broader efficacy and improved tolerability compared with first-generation drugs. However, their effectiveness in alleviating negative symptoms and cognitive deficits remains limited. Many antipsychotics provide only modest benefits while producing unwanted effects, such as sedation or extrapyramidal symptoms, which may further impair functioning. This highlights the need for novel pharmacological approaches capable of targeting the mechanisms underlying negative and cognitive symptoms.

Cariprazine is an orally active dopamine D3-preferring D3/D2 and serotonin 5-HT1A receptor partial agonist that improves both positive and negative symptoms and may also alleviate dysphoria in patients with schizophrenia.^1–3^ The unique pharmacological profile of cariprazine has led to the hypothesis that modulation of D3 receptor activity plays a critical role in cognitive and affective regulation, thereby contributing to improvements in negative and secondary symptoms. Preclinical data suggest that cariprazine can influence hippocampal gamma oscillations in vitro and may contribute to network stabilization via partial D3 receptor agonism.^4^^,^^5^ Such stabilization of local gamma-activity is likely to influence oscillatory dynamics across other frequency bands, given their strong cross-frequency interactions—for example, theta–gamma coupling in the hippocampus plays a critical role in working memory and cognitive integration.^6–8^ Since gamma oscillatory activity is often disrupted in schizophrenia and correlates with symptom severity,^9–11^ examining cortical oscillations in vivo during cariprazine treatment may provide crucial insights into its mechanism of action.

Electroencephalography is particularly well suited for this purpose, with 2 complementary approaches that it offers: (1) resting-state spectral analyses, which characterize the baseline dynamics of large-scale cortical networks, and (2) task-related event-related potentials (ERPs), which capture neural responses to specific cognitive or sensory events. We applied both approaches in the current study, but due to space limitations here, we report findings from the resting-state EEG analyses; results of task-related ERP measures will be presented separately.

Resting-state EEG spectral measures are especially promising for translational research, as they are non-invasive, repeatable, and cost-effective. They can be obtained without requiring complex tasks, allowing applicability even in severely ill patients. These measures have potential as predictive biomarkers (pretreatment indicators of likelihood of response) and response or monitoring biomarkers (treatment-related changes that track clinical improvement).

To explore these possibilities, the present investigation performed high-density (256-channel) EEG recordings in patients with schizophrenia initiating cariprazine treatment in a naturalistic clinical setting. We focused on resting-state activity by applying power spectral analyses across 5 major brain regions (frontal, central, parietal, temporal, occipital) in 6 frequency bands (delta, theta, alpha, beta, gamma1, gamma2).

To our knowledge, no prior human pharmaco-EEG study has examined spectral changes during cariprazine treatment. Of the 3 available preclinical EEG investigations, 2 were in vitro^4^^,^^5^; there was 1 in vivo study,^12^ conducted in rodents, which reported increases in theta and alpha power during wakefulness following cariprazine administration. In the wider context of human pharmaco-EEG research, it is noteworthy that antipsychotic medications—particularly second- and third-generation agents that are D2 and 5-HT2 receptor antagonists—are consistently associated with increases in theta power in schizophrenia.^13^^,^^14^

Besides broad regional analyses, we conducted targeted region of interest (ROI) analyses of gamma-activity in the dorsolateral prefrontal cortex (DLPFC), inferior frontal gyrus (IFG), and temporoparietal junction (TPJ)—regions repeatedly implicated in schizophrenia pathophysiology and associated with poor functional outcomes.^15^^,^^16^

The aims of this investigation were 2-fold: (1) to characterize the neurophysiological changes associated with cariprazine treatment in patients with schizophrenia and (2) to evaluate the potential of EEG spectral measures, particularly gamma oscillations, as biomarkers for treatment response, both for predictive and monitoring perspectives. Drawing on preclinical evidence for cariprazine and findings from human pharmaco-EEG studies,^13^^,^^14^ we expected that significant treatment-related changes (eg, increased theta power) would be observable in patients and would correlate with symptomatic improvement.

## Methods

### Study design and participants

This was a non-interventional investigation in patients with schizophrenia who were scheduled to receive cariprazine based on clinical judgment. Eligibility was restricted to patients treated in accordance with the latest approved Summary of Product Characteristics (SmPC) of cariprazine (marketed as Reagila in Hungary). Recruitment took place at the Department of Psychiatry and Psychotherapy, Semmelweis University (Budapest, Hungary). All participants provided written informed consent in line with the protocol, consistent with the Declaration of Helsinki and approved by the Scientific and Research Ethics Committee of the Medical Research Council in Hungary.

The study was a 12-week longitudinal investigation designed to obtain evaluable high-density EEG data from at least 25 patients with schizophrenia. The minimum sample size was chosen to guarantee 80% statistical power in order to detect significant associations (α = 0.05) between EEG data and symptom changes. The study consisted of 2 periods: a screening/baseline phase and a treatment phase. At screening, the diagnosis of schizophrenia was confirmed by a research psychiatrist based on the DSM criteria. Patients with significant medical illness, seizure disorder, substance abuse, or inability to provide informed consent were excluded. Screening procedures collected all data required to evaluate inclusion and exclusion criteria, which are detailed in the Appendix, Part 1. At baseline, prior to the first dose of cariprazine, participants underwent EEG investigation, as well as clinical and neurocognitive assessments, including the Positive and Negative Syndrome Scale (PANSS),^17^ administered as a semi-structured clinical interview by a clinician trained in the use of the instrument; these assessments were repeated during the treatment phase. Cariprazine dosing (1.5-6 mg/day) was determined by clinical judgment in this observational setting by the treating psychiatrist, independent of the EEG team (see Appendix, Part 2 for the schedule of medication dosing and cross-titration schedule for cariprazine).

### EEG recordings

EEG data were obtained by a 256-channel active-electrode system (Biosemi Inc, Amsterdam, The Netherlands) at a sampling rate of 512 Hz. High-density EEGs were recorded using a 256-channel BioSemi ActiveTwo system with an average reference, at a digitization rate of 512 Hz, applying a band-pass filter of 0.5-100 Hz. We applied a standard BioSemi 256-electrode headcap system (online source: http://www.biosemi.com/pics/cap_256_layout_medium.jpg). Data were stored and analyzed offline using the Electromagnetic Source Signal Imaging Suite, as well as the Statistical Analysis System (Version 9.4) software. During the EEG recording sessions, patients underwent both a resting-state EEG and a task-specific ERP assessment. For the resting EEG, participants sat in a dimly lit room and were instructed to remain still with their eyes closed for a duration of 3 minutes. For a detailed description of the preprocessing of the EEG signals, please refer to Appendix, Part 3.

### Statistical analyses

The frequency limits for the EEG band-power computation were the following: delta = 0.5-3.0 Hz; theta = 3.01-7.5 Hz; alpha = 7.51-12.0 Hz; beta = 12.01-30.0 Hz; gamma1 = 30.01-48 Hz; and gamma2 = 52.01-100 Hz. Band power was calculated by the summation of absolute power on frequency bins within the specified frequency range. Log10 transform was computed for the summed frequency band in order to achieve a better approximation of normal distribution compared to the raw data^18^ and to ensure methodological consistency with previous pharmaco-EEG studies.^19^

Band-power calculations were performed separately for each recording channel. The resulting log-transformed band-power values were then aggregated across 5 major brain regions—frontal, central, parietal, temporal, and occipital—using electrodes located in the left and right hemispheres, as well as along the midline. In addition to these predefined regional analyses, we conducted ROI analyses focusing on gamma activity both in the lower and higher frequency ranges (gamma1, gamma2). These ROI analyses targeted the IFG, the DLPFC, and the TPJ, as these areas are considered potential trait markers for schizophrenia and may exhibit neurophysiological alterations associated with poor functional outcomes.^15^^,^^16^^,^^20^ Electrode clusters in the BioSemi sensor system for the IFG, DLPFC, and TPJ have been described in our earlier investigation of resting-state gamma activity.^21^

Symptom severity was assessed with PANSS, a 30-item instrument comprising 7 positive, 7 negative, and 16 general psychopathology items.^17^ For detailed characterization, we applied a 5-factor model of PANSS including positive symptoms, negative symptoms, disorganized thought, uncontrolled hostility/excitement, and depression/anxiety.^22^ Change in symptom severity was computed as “baseline – endpoint”; therefore, a positive sign means improvement. In contrast, change in spectral power was computed as “endpoint – baseline”; hence, a positive value signifies an increase.

For descriptive statistical analyses to evaluate EEG power spectral changes at the Last Observation Carried-Forward (LOCF) endpoint, we performed a paired *t*-test for the respective time points and computed Cohen *D* values^23^ to characterize the magnitude of the effect sizes for the change. Inferential statistical analyses to examine concurrent and predictive associations between change in psychopathology and change in EEG spectral measures were based on random-regression Hierarchical Linear Model (HLM) analysis.^24^ Change in symptom severity, measured by the total and the 3 subscale scores of the PANSS scale, was used as a dependent variable in the analyses. Repeated measurements of spectral amplitudes (log-transformed μV^2^ units) across the various frequency bands were used as independent variables in the HLM. Time (visit number) served as a within-subject variable in the analyses.

In the analysis of predictive associations between pre-treatment EEG and response to treatment, we used symptom change as a categorical dependent variable, applying logistic regression. In the logistic regression analysis, at varying cut-off points of the baseline EEG-spectral measures, we computed the Receiver Operating Characteristic (ROC) curves^25^ to determine the true-positive rate (sensitivity) and false-positive rate (1-specificity) for predicting treatment response. The predictive accuracy was quantified by computing the Area Under the Receiver Operating Characteristic Curve (AUC). Besides the AUC-based evaluation, classification performance for distinguishing treatment responders from non-responders was assessed using a supervised machine learning approach—specifically, linear discriminant analysis—implemented via the SAS DISCRIM procedure. Responder status was designated as the categorical dependent variable, while baseline gamma-band activity from the 3 ROIs served as the predictor variables. Model generalizability was estimated using leave-one-out cross-validation (LOOCV), applied to data from the training dataset to test the robustness of the finding with respect to independent observations.

## Results

### Sample size and patient attrition

A total of 35 patients were enrolled in the investigation, of whom 27 completed at least 1 evaluable post-baseline assessment. Symptom severity, as measured by the total score of the PANSS scale, was similar in patients in the full analysis set (mean = 101.97, SD = 16.04) and in the EEG evaluable set (mean = 100.00, SD = 15.36). The symptom profiles based on the PANSS subscale scores were also similar (see Appendix, Part 4).

All descriptive and inferential analyses were conducted on the EEG-evaluable set (*n* = 27). Of these, 15 patients completed the week 6 visit, but the majority were lost to follow-up thereafter; given the small sample at visit 4 (*n* = 3), the LOCF endpoint included data through visit 3. Table 1 summarizes the demographic and clinical characteristics of the evaluable study sample. The mean age was 36.3 years (SD = 14.8), and the sample comprised 37% males (10/27) and 63% females (17/27). Male and female participants showed similar symptom presentation at baseline, as reflected in the PANSS total and subscale scores (see Appendix, Part 5). Mean daily cariprazine doses were 1.9 mg (SD = 0.7) at visit 2 and 3.1 mg (SD = 1.2) at visit 3.

**Table 1:** Basic demographic and clinical characteristics of the study sample (n = 27).

Measure			
Categorical variable (N, %)	N	%	Interquartile Range (25% - 75%)
Male/Female	10/17	37%/73%	n/a
Continuous variables	Mean	SD	
Age (years)	36.3	14.8	24.0 – 51.0
PANSS^a^			
TOT	100.000	15.36	91.0 – 112.0
POS	20.85	4.79	17.0 - 25.0
NEG	25.11	4.04	22.0 – 28.0
GEN	54.00	8.94	51.0 – 61.0

PANSS TOT, POS, NEG, and GEN refer to the total, positive, negative, and general psychopathology scores of the PANSS scale, respectively.

a PANSS=Positive and Negative Symptoms Scale.

### Changes in psychopathology during treatment

Baseline and follow-up symptom severity scores at weeks 2, 6, and the LOCF endpoint are presented in the Appendix (Part 6), based on PANSS total, subscale, and 5-factor scores. Table 2 summarizes the corresponding changes over time, indicating reductions in psychopathology relative to pre-treatment baseline at each assessment point. Positive values mean improvement, since change was computed by subtracting the value at a specific time point from the value at baseline. The *t*-statistics and *P*-values represent results of the paired *t*-tests and Type-I error rates, respectively. The last column of the table indicates the statistical effect size (Cohen *D*) for the change expressed in SD units. The respective statistics are provided separately for the total and subscale scores and for the 5 factors of the scale,^22^ namely, the Positive (Factor 1), Negative (Factor 2), Excitement (Factor 3), Cognitive (Factor 4), and the Depression factors (Factor 5). Following Cohen,^23^ effect size values of 0.3, 0.5, and 0.7 are interpreted, respectively, as small, medium, and large effect sizes. Values with *P* < .1 Type I error probability are highlighted in the table (see column labeled as *P*-value in the table); values with *P* < .05 are marked with an asterisk. To evaluate the effect size for the changes, Cohen *D* values in the medium-to-high effect size range (Cohen *D* > 0.4) are also highlighted in the table (see column labeled as Cohen *D* in Table 2).

**Table 2:** Changes in symptom severity over time based on PANSS scores (*n* = 27).

Measure	Visit (weeks)	Change Mean	Change SD	*t*-stat	*P*-value	Cohen *D*
PANSS TOT	2 weeks	5.370	9.552	2.92	.007^*^	0.563
	6 weeks	11.461	16.731	2.47	.029^*^	0.685
	LOCF endpoint	8.444	13.319	3.29	.003^*^	0.634
PANSS POS	2 weeks	1.111	3.320	1.74	.093	0.335
	6 weeks	2.538	5.092	1.80	.097	0.498
	LOCF endpoint	1.592	4.387	1.89	.069	0.363
PANSS NEG	2 weeks	1.037	2.967	1.82	.070	0.349
	6 weeks	4.000	3.937	3.66	.003^*^	1.016
	LOCF endpoint	2.666	3.573	3.88	.000^*^	0.746
PANSS GEN	2 weeks	3.185	6.051	2.74	.011^*^	0.526
	6 weeks	4.923	10.355	1.71	.112	0.475
	LOCF endpoint	4.148	8.113	2.66	.013^*^	0.511
PANSS Fact 1	2 weeks	0.888	3.273	1.41	.170	0.272
	6 weeks	1.692	4.607	1.32	.210	0.367
	LOCF endpoint	1.222	4.135	1.54	.136	0.296
PANSS Fact 2	2 weeks	0.962	3.168	1.58	.126	0.304
	6 weeks	3.307	3.614	3.30	.006^*^	0.915
	LOCF endpoint	2.333	3.269	3.71	.001^*^	0.714
PANSS Fact 3	2 weeks	0.296	1.659	0.93	.362	0.178
	6 weeks	0.384	2.142	0.65	.529	0.179
	LOCF endpoint	0.185	1.881	0.51	.613	0.098
PANSS Fact 4	2 weeks	1.222	1.928	3.29	.002^*^	0.634
	6 weeks	1.769	2.803	2.28	.042^*^	0.631
	LOCF endpoint	1.592	2.205	3.75	.001^*^	0.722
PANSS Fact 5	2 weeks	1.666	2.038	4.25	.000^*^	0.818
	6 weeks	1.769	4.186	1.52	.153	0.422
	LOCF endpoint	1.814	3.293	2.86	.008^*^	0.551

PANSS TOT, POS, NEG, and GEN refer to the total, positive, negative, and general psychopathology scores of the PANSS scale, respectively. Factors on the PANSS scale were computed according to Marder et al.^22^ PANSS factors 1 through 5 are Positive (Factor 1), Negative (Factor 2), Excitement (Factor 3), Cognitive (Factor 4), and the Depression factor (Factor 5), respectively. LOCF, Last Observation Carried Forward.

As shown in Table 2, significant improvements were observed for the LOCF endpoint for all measures except for the PANSS positive symptom scale, as well as the Positive symptom factor (Factor 1) and Excitement factor (Factor 3). The largest effect size was noted for the negative symptom scale (Cohen *D* = 0.746); medium-to-high effect sizes (Cohen *D* > 0.4) were observed for the statistically significant measures. Furthermore, the table indicates that significant improvements were already evident by week 2 for the PANSS total score, as well as the Cognitive (Factor 4) and Depression (Factor 5) factors. It is noteworthy that changes in symptom severity were closely associated with the baseline levels of psychopathology. In particular, the Pearson correlation between the total PANSS score at baseline and its change over time was 0.51 (*P* < .007), indicating that higher initial severity was associated with greater improvement.

In addition to quantifying clinical effects using Cohen *D* effect sizes, we also defined a dichotomous responder status. A 20% improvement threshold was applied, whereby patients showing at least a 20% reduction in PANSS total score over time were classified as responders. According to this criterion, 25.9% of the sample (7 of 27 patients) met the responder definition at the LOCF endpoint.

Finally, given that previous studies have raised the possibility^26^ that women may respond more favorably to antipsychotic treatment, we examined potential sex differences in treatment response by comparing improvements at the LOCF endpoint. The analysis revealed no significant differences between males and females in PANSS total, subscale, or 5-factor scores (see Appendix, Part 7).

### Changes over time in EEG power spectral measures

Our analyses examined changes in EEG spectral power from baseline to the treatment endpoint using the LOCF approach. Changes were calculated as the difference between endpoint and baseline values, with positive values indicating an increase. Table 3 summarizes these changes across 5 brain regions and 6 EEG frequency bands.

**Table 3:** Changes of EEG power spectral measures during the study at the LOCF endpoint.

EEG freq band	Brain area	Change Mean	Change SD	*t*-stat	*P*-value	Cohen *D*
delta	Frontal	0.077	1.033	0.39	.698	0.075
	Central	0.342	1.328	1.34	.191	0.257
	Parietal	0.702	1.761	2.07	.048^*^	0.398
	Occipital	0.530	1.221	2.26	.032^*^	0.434
	Temporal	0.389	1.623	1.25	.223	0.240
theta	Frontal	0.070	1.037	0.35	.728	0.067
	Central	0.430	1.303	1.72	.097	0.330
	Parietal	0.827	1.693	2.54	.017^*^	0.488
	Occipital	0.619	1.205	2.67	.012^*^	0.514
	Temporal	0.433	1.539	1.47	.154	0.281
alpha	Frontal	0.123	1.055	0.61	.548	0.116
	Central	0.448	1.402	1.66	.108	0.319
	Parietal	0.810	1.828	2.30	.029^*^	0.443
	Occipital	0.623	1.382	2.34	.026^*^	0.451
	Temporal	0.346	1.583	1.14	.265	0.219
beta	Frontal	0.020	0.843	0.12	.901	0.024
	Central	0.322	1.080	1.55	.133	0.298
	Parietal	0.591	1.435	2.14	.041^*^	0.412
	Occipital	0.462	1.026	2.34	.027^*^	0.450
	Temporal	0.239	1.295	0.96	.345	0.184
gamma1	Frontal	0.141	0.827	0.89	.383	0.170
	Central	0.392	1.023	1.99	.057	0.383
	Parietal	0.481	1.305	1.92	.066	0.368
	Occipital	0.441	1.018	2.25	.033^*^	0.433
	Temporal	0.263	1.259	1.08	.288	0.208
gamma2	Frontal	0.230	0.886	1.35	.188	0.259
	Central	0.400	1.086	1.92	.066	0.368
	Parietal	0.372	1.288	1.50	.145	0.289
	Occipital	0.380	1.107	1.78	.086	0.342
	Temporal	0.244	1.375	0.93	.363	0.178

Asterisk (^*^) indicates *P* < .05. LOCF, Last Observation Carried Forward.

Specifically, Table 3 presents the results of paired *t*-tests, including *t*-statistics, corresponding *P*-values, and effect sizes (Cohen *D*, expressed in SD units). *P*-values with <.10 are highlighted, and corrections for multiple comparisons were applied using the False Discovery Rate (FDR) method separately for each brain region. Nominally significant results that remained significant after FDR correction are marked with an asterisk. Overall, mean spectral power values showed a consistent tendency to increase across brain regions. Statistically significant increases were observed in the parietal and occipital regions, with the exception of the gamma1 and gamma2 bands in the parietal region and the gamma1 band in the occipital region. For regions with significant changes, effect sizes were generally in the medium range (Cohen *D* > 0.4).

In addition to the primary analyses pooling signals across hemispheres, we conducted exploratory analyses to assess potential lateralized effects. Effect sizes (Cohen *D*) for spectral power changes at the LOCF endpoint were calculated separately for the right and left hemispheres. As shown in the Appendix, Part 8, effect sizes were slightly higher in the right hemisphere, but differences between hemispheres were minimal. Consequently, subsequent analyses focused on overall brain regions rather than hemisphere-specific effects.

### Concurrent association between change in psychopathology and change in power spectral measures across brain regions

In this region-wise topographical analysis, using general linear model analysis, we investigated whether changes in PANSS total scores were associated with the spectral power changes in 5 brain regions. Our findings presented in Table 4 revealed that increase in theta activity alongside a decrease in delta activity during treatment was associated with changes in PANSS total scores across all 5 topographical areas, with the exception of the theta-activity in the frontal area where the association was marginally significant (*P* = .059). The sign of the regression coefficients indicates that the changes (ie, increase in theta and simultaneous decrease in delta power during treatment) in the 2 frequency bands were linked to an improvement in the total score of the PANSS scale.

**Table 4:** Association between change in PANSS total score and power spectral measures at LOCF endpoint.

Brain region	Frequency	Type III SS	Estimate	SE	*t*	*P*
Frontal	Delta	1466.346	-19.436	7.675	-2.53	.019^*^
	Theta	911.834	23.326	11.68	2.00	.059
	Alpha	0.39	0.383	9.265	0.04	.968
	Beta	306.381	-18.376	15.875	-1.16	.260
	Gamma1	71.762	9.768	17.435	0.56	.581
	Gamma2	72.656	5.13	9.101	0.56	.579
Central	Delta	1986.971	-22.302	6.781	-3.29	.004^*^
	Theta	1765.332	30.856	9.954	3.10	.005^*^
	Alpha	4.931	-1.315	8.029	-0.16	.871
	Beta	443.798	-23.382	15.043	-1.55	.135
	Gamma1	174.589	12.783	13.112	0.97	.341
	Gamma2	48.884	3.538	6.858	0.52	.611
Parietal	Delta	1184.712	-15.803	6.574	-2.40	.026^*^
	Theta	1771.880	26.386	8.976	2.94	.008^*^
	Alpha	49.962	-3.939	7.979	-0.49	.627
	Beta	39.104	-7.255	16.612	-0.44	.667
	Gamma1	23.918	-4.484	13.130	-0.34	.736
	Gamma2	72.200	4.835	8.147	0.59	.559
Temporal	Delta	1195.466	-16.059	6.715	-2.39	.026^*^
	Theta	1567.804	26.709	9.752	2.74	.012^*^
	Alpha	95.292	-6.925	10.256	-0.68	.507
	Beta	79.004	-8.381	13.632	-0.61	.545
	Gamma1	24.896	4.415	12.793	0.35	.733
	Gamma2	9.240	1.388	6.600	0.21	.836
Occipital	Delta	1688.653	-22.373	7.717	-2.90	.009^*^
	Theta	1632.728	33.211	11.651	2.85	.010^*^
	Alpha	77.405	-7.003	11.283	-0.62	.542
	Beta	48.965	-9.126	18.486	-0.49	.627
	Gamma1	16.311	3.905	13.704	0.28	.779
	Gamma2	32.557	2.898	7.198	0.40	.691

a: Type III SS = Type III Sum of Squares; b: Estimate = regression estimate from the HLM; c: SE = standard error of the estimate.

^*^ *P* < .05. LOCF, Last Observation Carried Forward.

We next examined whether the associations observed for the PANSS total score extended to the 3 subscales. Consistent with the overall findings, changes in spectral power were significantly associated with improvements in positive symptoms (*P* < .05 across analyses). Similar trends were observed for general psychopathology and negative symptoms, although these did not consistently reach statistical significance across brain regions.

To assess the timing of these effects, we further tested whether the relationship between spectral power and symptom change could be detected at week 2. As shown in detail in Appendix, Part 9, concurrent increases in theta activity and decreases in delta activity were significantly associated with reductions in PANSS total scores in 3 regions (central, parietal, occipital). In contrast, the association was weaker in the frontal (theta, *P* = .105) and temporal (delta, *P* = .115) regions.

### Predictive relationships between spectral power and symptom change

We applied 2 complementary approaches to examine whether baseline spectral power predicted subsequent improvement in symptom severity during treatment. The first consisted of region-wise analyses across 5 broad topographical areas, while the second focused on a predefined ROI analysis targeting gamma-band activity in selected cortical regions (see “Methods”).

### Region-wise analyses

To test whether baseline spectral power predicted treatment response, logistic regression models were fitted with the change in PANSS total score at the LOCF endpoint as the dependent variable. Independent variables included baseline spectral power across 6 frequency bands in each of the 5 predefined brain regions. Separate analyses were performed for each region. These models revealed no significant associations between baseline spectral power and changes in symptom severity (*P* > .1 in all analyses).

### ROI analyses

In a second set of analyses, we investigated gamma-band activity in 3 a priori ROIs, including the DLPFC, TPJ, and IFG. Logistic regressions were conducted with change in PANSS total score as the dependent variable and baseline spectral power within these ROIs as predictors. Given that the baseline PANSS total score was itself associated with symptom improvement, it was included as a covariate in additional models.

When considering EEG variables alone, baseline gamma1 activity showed a marginal association with treatment outcome at the LOCF endpoint (Likelihood-Ratio χ^2^ = 2.9952, *P* = .0835), while gamma2 activity was significantly associated (Likelihood-Ratio χ^2^ = 7.3941, *P* = .006). When baseline symptom severity was included in the models, both gamma bands showed robust associations: gamma1 (Likelihood-Ratio χ^2^ = 13.9244, *P* = .003) and gamma2 (Likelihood-Ratio χ^2^ = 20.223, *P* = .0002).

To characterize the direction and magnitude of these associations, we compared responders and non-responders at the LOCF endpoint. Descriptive statistics are presented in Table 5 for the 2 gamma bands. Across all regions, non-responders—defined as patients with <20% improvement in PANSS total score—exhibited numerically higher baseline gamma power than responders. Effect size estimates (Cohen *D*) indicated medium-to-large differences in the IFG, medium differences in the DLPFC, and small differences in the TPJ.

**Table 5:** Descriptive statistics and Cohen *D* effect size values for gamma spectral measures across the 3 brain regions for non-responders (*n* = 20) and responders (*n* = 7) at LOCF endpoint.

Frequency band	Region	Non-responder mean	Non-responder SD	Responder mean	Responder SD	Cohen *D*
Gamma1	DLPFC	-0.401	1.937	-1.071	1.241	0.45
	TPJ	-0.594	2.474	-0.746	1.483	0.08
	IFG	0.436	2.127	-0.467	1.105	0.56
Gamma2	DLPFC	-3.355	2.336	-4.481	1.625	0.59
	TPJ	-3.157	3.083	-3.711	1.244	0.26
	IFG	-2.407	2.415	-3.714	1.401	0.69

Abbreviations: DLPFC, dorsolateral prefrontal cortex; IFG, inferior frontal gyrus; LOCF, Last Observation Carried Forward; TPJ, temporoparietal junction.

### Evaluation of predictive performance using receiver operating characteristic curve analysis

Besides Cohen *D* values, we also computed the ROC curves and determined the AUC to determine the predictive ability of the gamma-activity measures to distinguish between responders and non-responders. Theoretically, the AUC values range from 0.5 (no discrimination) to 1.0 (perfect discrimination), with values of 0.7-0.8 considered acceptable, 0.8-0.9 excellent, and >0.9 outstanding.^25^

**Figure 1:**
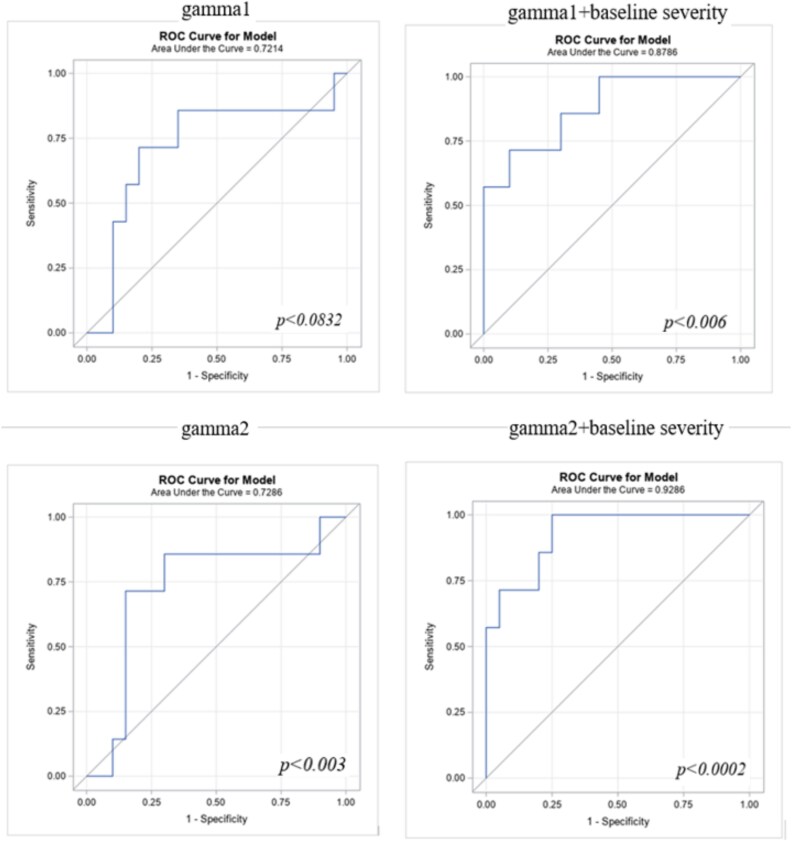
ROC curves for baseline EEG spectral measures, depicting sensitivity (true positive rate) and 1-specificity (false positive rate) in predicting treatment response at study endpoint for the gamma1 (top) and gamma2 (bottom) bands. The curves and the corresponding AUC values illustrate predictive performance of gamma measures alone (left panels) and combined with baseline symptom-severity (right panels). Adding baseline symptom-severity improved model performance, increasing AUC from ~0.72 to >0.85. The strongest effect was observed for high-frequency gamma, where the AUC exceeded 0.92.

Figure 1 shows ROC curves for baseline EEG spectral measures, depicting sensitivity (true-positive rate) and 1-specificity (false-positive rate) in predicting treatment response at the endpoint of the investigation for the gamma1 (top) and gamma2 (bottom) bands. The curves and corresponding AUC values illustrate the predictive performance of gamma measures alone (left panels) and combined with baseline symptom severity (right panels).

Receiver Operating Characteristic analyses for both gamma1 (top) and gamma2 (bottom) bands showed predictive associations with responder status, with AUC values ranging from 0.72 to 0.92. Three of the 4 models were statistically significant, with 1 marginally significant result (gamma1: *P* = .0832). Adding baseline symptom severity improved model performance, increasing the AUC from ~0.72 to >0.85. The strongest effect was observed for high-frequency gamma, where the AUC exceeded 0.92, reflecting outstanding discriminative accuracy.

### Accuracy of responder classification

Beyond ROC curve analysis, we assessed the ability of baseline gamma activity in predefined ROIs to classify responders and non-responders. Classification was performed using linear discriminant analysis, with responder status at the LOCF endpoint as the dependent variable and gamma activity from the 3 ROIs as predictors. Separate analyses were conducted for the gamma1 and gamma2 frequency bands at both week 2 and the LOCF endpoint.

Model performance was evaluated using LOOCV, with classification accuracies reported to reflect performance on subjects independent of the training set. Results are summarized in Table 6. Responders and non-responders at LOCF were distinguished with >70% accuracy based on baseline gamma activity, with mean classification error rates of 26.8% (gamma1) and 24.3% (gamma2). As shown in Table 6, adding baseline symptom severity slightly reduced performance, increasing error rates to 31.5% (gamma1) and 34.0% (gamma2).

**Table 6:** Classification accuracy of responders vs. non-responders based on gamma spectral measures with leave-one-out cross-validation (LOOCV).

Frequency band	Predictors	Error rate (%)	Accuracy (%)
Gamma1	EEG only	26.8	73.2
	EEG + Baseline PANSS	31.5	68.5
Gamma2	EEG only	24.3	75.7
	EEG + Baseline PANSS	34.0	66.0

Abbreviations: LOOCV, Leave-One-Out Cross-Validation; PANSS, Positive and Negative Syndrome Scale.

Beyond overall classification accuracy, we assessed prediction confidence by examining the estimated probabilities of correct class membership for responders and non-responders. Detailed results for gamma1 and gamma2 are provided in the Appendix, Part 10. Overall, responders were identified as responders with a high predicted probability, particularly in the gamma2 range (median = 70%). In contrast, non-responders were (mis-)classified as responders with notably low probability across both gamma frequency ranges, with median predicted probabilities of only 7% and 4% for the gamma1 and gamma2 ranges, respectively.

## Discussion

This exploratory high-density EEG study provides novel insights into the neurophysiological correlates of symptom improvement in schizophrenia during treatment with cariprazine, a dopamine D3/D2 receptor partial agonist with preferential affinity for D3 receptors. However, beyond its dopaminergic profile, cariprazine also exhibits partial agonism at 5-HT1A receptors and antagonism at 5-HT2A and 5-HT2B receptors.^27^ Given that 5-HT1A receptor agonism has been associated with mood and cognitive regulation,^28^ the observed neurophysiological effects may reflect the integrated influence of both dopaminergic and serotonergic mechanisms rather than dopaminergic activity alone.

Consistent with earlier clinical findings,^2^^,^^3^ our results demonstrated significant improvement in psychopathology, reflected by reductions in PANSS total and subscale scores. Improvements were detectable as early as week 2 and continued during the follow-up period, with the strongest and most consistent effects observed for negative and general symptoms. This pattern of clinical response is particularly noteworthy, as negative symptoms and associated cognitive deficits are major determinants of functional disability in schizophrenia and remain insufficiently addressed by most currently available antipsychotics.^29^ Overall, our findings align with previous trials demonstrating the efficacy of cariprazine across a wide range of symptoms, including the negative symptom domain in schizophrenia,^2^^,^^3^ as well as with prior evidence indicating no significant sex differences in antipsychotic response.^26^

### Spectral changes and clinical improvement

Beyond symptomatic improvement, cariprazine treatment was associated with increased spectral power, with the largest effect sizes in the theta band and the alpha band, consistent with prior pharmaco-EEG studies showing that antipsychotics generally induce background slowing, albeit with some variability across agents.^14^^,^^19^^,^^30^^,^^31^ Notably, our results also replicate findings from the only available in vivo preclinical EEG study of cariprazine, which reported increased theta and alpha power in wakefulness in rats during treatment.^12^ The convergence between rodent and human findings underscores the cross-species translatability of EEG spectral measures, supporting their utility as biomarkers in drug development.

The spectral changes that we found during treatment were associated with symptom improvement, most prominently an increase in theta activity accompanied by a relative decrease in delta activity. This theta–delta shift was the most robust in the parietal and occipital regions and was significantly associated with reductions in symptom severity. The directionality of these effects—greater theta and reduced delta power predicting greater improvement—suggests normalization of cortical dynamics. In schizophrenia, excessive delta activity is commonly interpreted as a marker of cortical slowing and reduced network efficiency,^32^ whereas enhanced theta activity may reflect improved integrative processing and synchronization.^13^^,^^14^

Together, these results imply that cariprazine treatment may facilitate a functional rebalancing of large-scale cortical networks, which, in turn, contributes to symptomatic relief. It is also noteworthy that the regions where the most prominent increases in theta activity were found (ie, parietal and occipital areas) play a central role in sensory integration, visual processing, and attentional control^33^^,^^34^—domains that are often impaired in schizophrenia.^35^ The observed increases in spectral power in these regions may therefore reflect improved integration of sensory input and enhanced attentional resource allocation. The fact that these changes occurred in a resting-state paradigm underscores the intrinsic modulation of baseline network function by cariprazine, independent of explicit cognitive engagement. Overall, the finding that neurophysiological associations were detectable between treatment response and resting-EEG spectral measures—which have an established role for cross-species translation during drug discovery^36^^,^^37^—further highlights the promise of these measures as a translational tool for biomarker development.

### Role of resting gamma activity in regions of interest

We placed particular emphasis on examining gamma-band oscillations due to their pivotal role in synchronizing cortical networks, a process that is consistently disrupted in schizophrenia. Aberrant gamma activity has been robustly linked to deficits in perception, working memory, and higher cognitive functions, as well as with the positive and negative symptom domains seen in the illness.^9^^,^^10^ Accordingly, our ROI analyses centered on the DLPFC, TPJ, and IFG—regions critically involved in these cognitive operations and viewed as central to schizophrenia pathophysiology.^38^

Region of interest analyses revealed that non-responders consistently showed higher baseline gamma-activity than responders. Logistic regression and ROC analyses confirmed that baseline gamma—especially high-frequency (gamma2)—significantly predicted treatment response, with associations remaining robust after controlling for baseline symptom severity. Effect size estimates indicated medium-to-large differences in the IFG, medium effects in the DLPFC, and smaller effects in the TPJ, suggesting that frontal and fronto-temporal regions may play a key role in distinguishing treatment outcomes.

The functional relevance of these regions reinforces their potential utility as predictive biomarkers. The DLPFC is a critical hub for executive function and working memory, both commonly impaired in schizophrenia.^39^ The IFG is involved in response inhibition and language processing,^40^^,^^41^ while the TPJ plays a central role in social cognition and self-other processing.^42^ Aberrant gamma activity in these areas may therefore represent a neural substrate of the cognitive and social deficits that hinder functional recovery in schizophrenia. Identifying patients with elevated resting gamma in these regions as less likely to respond to treatment could inform stratification strategies in both clinical trials and clinical practice.

### Predictive value of gamma activity and clinical utility

ROC analyses demonstrated that baseline gamma-activity was a good predictor of responder status, with AUC values ranging from acceptable to outstanding (0.72-0.92). The predictive power was further enhanced when baseline symptom severity was included as an additional predictor, with AUC values exceeding 0.92 for the gamma2 frequency range.

The classification analyses provided additional support for these findings, with linear discriminant analysis and LOOCV achieving >70% accuracy in distinguishing responders from non-responders. Error rates were as low as 24%-27%, reflecting a high degree of predictive reliability. Importantly, LOOCV provides a stringent test of model generalizability by validating predictions on independent observations, thereby reducing the risk of overfitting. That such high classification accuracy was achieved with a relatively modest sample size underscores the potential of gamma-band EEG measures as clinically useful biomarkers.

In practice, this level of predictive accuracy could enable early identification of patients most likely to benefit from cariprazine, supporting personalized treatment decisions and optimizing therapeutic outcomes. More broadly, in the context of other pharmaco-EEG findings,^13^^,^^14^ these EEG alterations may extend beyond cariprazine. and could also serve—at least in part—as biomarkers of treatment response across other antipsychotics, reflecting shared mechanisms of network modulation in schizophrenia.

### Clinical and translational implications

Taken together, these findings support the use of EEG spectral measures as translational biomarkers in schizophrenia. Resting-state EEG provides a scalable and non-invasive index of cortical network function, and in this study, both baseline predictors (eg, gamma activity in the DLPFC, TPJ, and IFG) and treatment-related changes (theta and delta shifts in posterior brain regions) were associated with symptomatic improvement. Clinically, such measures could enable stratification in trials, improving statistical power by reducing heterogeneity, and inform early treatment decisions by identifying likely responders. As monitoring tools, spectral EEG indices may offer objective markers of therapeutic response to complement clinical ratings, and, with further validation, could serve as surrogate endpoints to accelerate drug development targeting network-level dysfunction in schizophrenia. Finally, our findings highlight the cross-species translatability of EEG spectral measures, reinforcing their value as biomarkers for antipsychotic drug development.

### Limitations and future directions

Despite these promising results, several limitations warrant consideration. The sample size was modest, and attrition limited the availability of data at later time points. While LOOCV reduces the risk of overfitting, replication in larger and independent cohorts will be necessary to confirm the robustness and generalizability of these findings. In addition, while resting-state EEG offers practical advantages, future studies should also integrate task-based paradigms to capture dynamic aspects of network dysfunction related to specific cognitive processes. Furthermore, longitudinal studies tracking both clinical and neurophysiological trajectories over extended periods are important to further clarify the temporal dynamics of treatment-related changes. Finally, a potential limitation of the study is the relatively low response rate, which may be attributable to the lower cariprazine doses used. Observational data indicate that, in clinical practice, many patients are maintained on approximately 3 mg/day to balance efficacy and tolerability.^43^ Future investigations employing higher doses may help to further elucidate dose–response relationships in pharmaco-EEG measures.

## Supplementary Material

Cariprazine_humanEEG_Appendix_pyag008

## Data Availability

The datasets presented in this article are not readily available because they are part of a bigger study dataset, where the analyses are ongoing. While the full datasets can therefore not be shared, all statistical analyses and data outputs generated for the present study/publication can be requested from the authors.
