# Supplementary material for: High-density EEG study in patients with schizophrenia treated with cariprazine
Source: Int J Neuropsychopharmacol. 2026 Mar 9;29(4):pyag008. doi: 10.1093/ijnp/pyag008 (PMC13056714; doi:10.1093/ijnp/pyag008)
Supplement: Cariprazine_humanEEG_Appendix_pyag008 [file cariprazine_humaneeg_appendix_pyag008.doc]

# Appendix

# Appendix, Part 1: Study population (inclusion & exclusion criteria)

| **Inclusion Criteria**  - Diagnosis: DSM-5 schizophrenia.  - Age: 18-65 years.  - Sex: Male, or non pregnant female subjects  - General Health: No relevant abnormality that may influence the EEG activity.  - Concomitant standing or prn medications (except other antipsychotics and those contraindicated in the respective package inserts) are permitted, if they were present at a stable dose for at least 6 weeks prior to the start of initial treatment with study medication.  - Schizophrenia diagnosed at least one year before the screening  - Clinical judgment by the investigator that treatments with cariprazine is warranted due to suboptimal clinical outcome despite previous treatments  - Patient is judged capable of understanding all relevant risks and potential benefits of the study and provides informed consent.  **Exclusion criteria**  - Any potential contraindication for Cariprazine, as indicated in the SmPC.  - History of positive hepatitis B surface antigen.  - Any abnormal laboratory test that might interfere with the EEG, according to the investigator's judgement.  - A history of significant head injury/trauma, as defined by:  - loss of consciousness for more than 1 hour  - clear cognitive sequelae of head injury  - history of cognitive rehabilitation following the injury  - alcohol or substance dependence within the past 12 months or abuse within the past 3 months. Any subject with positive urine toxicology or alcohol use that is considered abnormal at baseline.  - Clinically significant suicidal or homicidal behavior or attempts within past 6 months.  - Any subject judged by the investigator to present a danger to self or others.  - Presence of any unstable or untreated medical disorder.  - Any subject who is judged by the investigator to be unable or unlikely to comply with all study requirements, including adherence with prescribed medication regimen.  - Pregnant (excluded by pregnancy test) or breast-feeding women. |
| --- |

# Appendix, Part 2: Schedule of medication dosing and cross-titration schedule for cariprazine

Transition from prestudy antipsychotic treatment to cariprazine followed a gradual cross-titration schedule. During the first week, the previous antipsychotic was tapered while cariprazine was initiated at 1.5 mg/day and titrated upward based on clinical response and tolerability. Dosing adjustments were permitted according to clinical status, including side effect profile. Because anticholinergic medications may interfere with assessments, their use was prohibited within 48 hours prior to testing. If intermittent anticholinergic treatment (≤3 consecutive days) was required, study assessments were postponed until at least 48 hours after the last dose. Likewise, to minimize the influence of sedation, assessments were postponed for at least 24 hours after the last administration of any medication for agitation

# Appendix, Part 3: Pre-processing of the EEG signals

EEG was re-referenced off-line to the common average potential and filtered between 0.5 and 100 Hz using zero-phase shiftforward and reverse IIR Butterworth-filter. Additionally, the signal was filtered using the 48–52 Hz Parks-McClellan stop-band Notch filter. The notch filter was used to remove any potential electric-interference from the 50 Hz line. Artifacts due to blinks and eye movements were removed manually and with the electrooculography artifact removal procedure. Epoch selection for the analyses was conducted manually, as well as applying automatic artifact rejection criteria. Resting state EEG data analysis was performed on 2.5 min of artifact-free EEG data, according to its reliability in resting-state EEG power spectra. On the corrected EEG data FFT was performed to extract absolute power based on Welch’s method, using a 2-s, 75% overlap window and 0.5 Hz frequency bin resolution.

# Appendix, Part 4: Symptom severity at baseline in the full sample and in the evaluable set

| PANSS measure | Full sample | | Evaluable by protocol | |
| --- | --- | --- | --- | --- |
|  | Mean | SD | Mean | SD |
| Total score | 101.968 | 16.042 | 100.000 | 15.355 |
| Positive  subscale | 21.419 | 4.801 | 20.852 | 4.792 |
| Negative subscale | 25.290 | 4.068 | 25.111 | 4.041 |
| General psychopathology | 55.225 | 9.387 | 54.000 | 8.944 |

# Appendix, Part 5: Symptom severity at baseline by gender

| PANSS^a^ measure | Male (N=10) | | Female (N=17) | | Group Comparison^b^  (Male – Female) | | |
| --- | --- | --- | --- | --- | --- | --- | --- |
|  | Mean | SD | Mean | SD | Difference: Estimate (SE) | t | p |
| Total score | 100.600 | 17.031 | 99.647 | 14.820 | 0.953 (0.238) | 0.15 | 0.880 |
| Positive  subscale | 20.300 | 6.001 | 21.176 | 4.096 | -0.877 (1.940) | -0.45 | 0.655 |
| Negative subscale | 26.100 | 2.807 | 24.529 | 4.598 | 1.571 (1.612) | 0.97 | 0.339 |
| General psychopathology | 54.100 | 10.429 | 53.941 | 8.250 | 0.159 (3.635) | 0.04 | 0.966 |

^a^: PANSS=Positive and Negative Symptoms Scale

^b^: Group comparisons were based on Analysis of Variance

# Appendix, Part 6: Baseline and follow-up symptom severity scores at weeks 2, 6, and the LOCF endpoint in the sample (n=27)

| **Measure** | **Visit (weeks)** | **Mean** | **SD** |
| --- | --- | --- | --- |
| **PANSS TOT** | 0 (baseline) | 100.000 | 15.355 |
|  | 2 weeks | 94.629 | 15.551 |
|  | 6 weeks | 89.461 | 16.800 |
|  | LOCF end point | 92.423 | 14.798 |
| **PANSS POS** | 0 (baseline) | 20.852 | 4.793 |
|  | 2 weeks | 19.741 | 3.928 |
|  | 6 weeks | 18.615 | 4.253 |
|  | LOCF end point | 19.259 | 3.558 |
| **PANSS NEG** | 0 (baseline) | 25.111 | 4.041 |
|  | 2 weeks | 24.074 | 3.562 |
|  | 6 weeks | 21.769 | 4.245 |
|  | LOCF end point | 22.444 | 3.445 |
| **PANSS GEN** | 0 (baseline) | 54.000 | 8.944 |
|  | 2 weeks | 50.814 | 9.499 |
|  | 6 weeks | 49.076 | 9.322 |
|  | LOCF end point | 49.851 | 8.506 |
| **PANSS Factor 1** | 0 (baseline) | 17.629 | 4.342 |
|  | 2 weeks | 16.740 | 3.358 |
|  | 6 weeks | 15.923 | 3.475 |
|  | LOCF end point | 16.407 | 3.066 |
| **PANSS Factor 2** | 0 (baseline) | 20.925 | 3.931 |
|  | 2 weeks | 19.962 | 3.106 |
|  | 6 weeks | 18.307 | 3.705 |
|  | LOCF end point | 18.592 | 2.977 |
| **PANSS Factor 3** | 0 (baseline) | 9.148 | 2.248 |
|  | 2 weeks | 8.851 | 2.125 |
|  | 6 weeks | 8.538 | 1.761 |
|  | LOCF end point | 8.962 | 1.720 |
| **PANSS Factor 4** | 0 (baseline) | 16.703 | 2.446 |
|  | 2 weeks | 15.481 | 2.259 |
|  | 6 weeks | 15.076 | 2.628 |
|  | LOCF end point | 15.111 | 2.189 |
| **PANSS Factor 5** | 0 (baseline) | 14.740 | 3.241 |
|  | 2 weeks | 13.074 | 3.221 |
|  | 6 weeks | 12.769 | 3.031 |
|  | LOCF end point | 12.925 | 3.037 |

Note: PANSS TOT, POS, NEG, and GEN refer to the total, positive, negative, and general psychopathology scores of the PANSS scale, respectively. Factors on the PANSS scale were computed according to Marder et al. (1997). PANSS factors 1 through 5 are Positive (Factor 1), Negative (Factor 2), Excitement (Factor 3), Cognitive (Factor 4), and the Depression factor (Factor 5), respectively. LOCF = Last Observation Carried Forward.

# Appendix, Part 7: Changes in symptom severity in males and females at LOCF endpoint

| PANSS^a^ measure | Male (N=10) | | Female (N=17) | | Group Comparison^b^  (Male – Female) | | |
| --- | --- | --- | --- | --- | --- | --- | --- |
|  | Mean | SD | Mean | SD | Difference: Estimate (SE) | t | p |
| Total score | 8.900 | 13.152 | 8.176 | 13.812 | 0.725 (5.411) | 0.13 | 0.895 |
| Positive  subscale | 1.400 | 4.033 | 1.705 | 4.700 | -0.306 (1.782) | -0.17 | 0.865 |
| Negative subscale | 3.200 | 2.936 | 2.352 | 3.951 | 0.847 (1.442) | 0.59 | 0.562 |
| General psychopathology | 4.200 | 8.854 | 4.117 | 7.928 | 0.082 (3.297) | 0.02 | 0.980 |
| Factor 1^c^ | 1.000 | 3.431 | 1.352 | 4.595 | -0.353 (1.679) | -0.21 | 0.835 |
| Factor 2 | 2.900 | 2.846 | 2.000 | 3.535 | 0.900 (1.312) | 0.68 | 0.501 |
| Factor 3 | 0.400 | 1.955 | 0.058 | 1.886 | 0.341 (0.762) | 0.45 | 0.658 |
| Factor 4 | 0.800 | 2.201 | 2.058 | 2.135 | -1.259 (0.861) | -1.46 | 0.156 |
| Factor 5 | 1.600 | 3.405 | 1.941 | 3.325 | -0.341 (1.337) | -0.26 | 0.801 |

Note:

^a^: PANSS=Positive and Negative Symptoms Scale

^b^: Group comparisons were based on Analysis of Variance

^c^: Factors on the PANSS scale were computed according to Marder et al. (1997). PANSS factors 1 through 5 are Positive (Factor 1), Negative (Factor 2), Excitement (Factor 3), Cognitive (Factor 4), and the Depression factor (Factor 5), respectively. LOCF = Last Observation Carried Forward.

# Appendix, Part 8: Statistical effect size (Cohen D) for LOCF changes in spectral power in the right and left hemispheres throughout the study, along with the effect size difference between the two hemispheres

| EEG freq  band | brain area | Cohen-D  Right hemisphere | Cohen-D  Left hemisphere | Cohen-D diff.  Right – Left |
| --- | --- | --- | --- | --- |
| delta | frontal | 0.018 | 0.122 | -0.104 |
|  | central | 0.255 | 0.219 | 0.036 |
|  | parietal | **0.426** | 0.317 | 0.109 |
|  | occipital | **0.456** | 0.386 | 0.070 |
|  | temporal | 0.102 | 0.307 | -0.205 |
| theta | frontal | -0.022 | 0.155 | -0.177 |
|  | central | 0.355 | 0.251 | 0.104 |
|  | parietal | **0.496** | 0.381 | 0.115 |
|  | occipital | **0.500** | **0.413** | 0.087 |
|  | temporal | 0.184 | 0.312 | -0.128 |
| alpha | frontal | 0.013 | 0.207 | -0.194 |
|  | central | 0.335 | 0.242 | 0.093 |
|  | parietal | **0.411** | 0.359 | 0.052 |
|  | occipital | **0.448** | 0.414 | 0.034 |
|  | temporal | 0.123 | 0.255 | -0.132 |
| beta | frontal | -0.101 | 0.158 | -0.259 |
|  | central | 0.327 | 0.208 | 0.119 |
|  | parietal | 0.382 | 0.319 | 0.063 |
|  | occipital | **0.461** | 0.391 | 0.070 |
|  | temporal | 0.016 | 0.256 | -0.240 |
| gamma1 | frontal | 0.041 | 0.265 | -0.224 |
|  | central | **0.400** | 0.294 | 0.106 |
|  | parietal | 0.316 | 0.308 | 0.008 |
|  | occipital | **0.435** | 0.354 | 0.081 |
|  | temporal | 0.012 | 0.288 | -0.276 |
| gamma2 | frontal | 0.161 | 0.315 | -0.154 |
|  | central | 0.348 | 0.326 | 0.022 |
|  | parietal | 0.227 | 0.263 | -0.036 |
|  | occipital | 0.358 | 0.286 | 0.072 |
|  | temporal | -0.040 | 0.304 | -0.344 |

# Appendix, Part 9: Association between PANSS total score and power spectral measures in the five brain areas during the first 2 weeks in the study

| brain region | frequency | Type III SS^a^ | estimate^b^ | se^c^ | t | p |
| --- | --- | --- | --- | --- | --- | --- |
| frontal | delta | 609.938 | -12.535 | 5.47 | -2.29 | **0.032** |
| frontal | theta | 333.469 | 14.106 | 8.325 | 1.69 | 0.105 |
| frontal | alpha | 0.018 | 0.081 | 6.604 | 0.01 | 0.99 |
| frontal | beta | 52.422 | -7.601 | 11.315 | -0.67 | 0.509 |
| frontal | gamma1 | 2.209 | 1.714 | 12.427 | 0.14 | 0.892 |
| frontal | gamma2 | 31.885 | 3.399 | 6.487 | 0.52 | 0.606 |
| central | delta | 755.834 | -13.755 | 4.943 | -2.78 | **0.011** |
| central | theta | 795.199 | 20.71 | 7.256 | 2.85 | **0.009** |
| central | alpha | 8.347 | -1.711 | 5.853 | -0.29 | 0.773 |
| central | beta | 136.456 | -12.965 | 10.966 | -1.18 | 0.25 |
| central | gamma1 | 20.047 | 4.332 | 9.559 | 0.45 | 0.655 |
| central | gamma2 | 36.835 | 3.071 | 5.0 | 0.61 | 0.546 |
| parietal | delta | 412.876 | -9.329 | 4.478 | -2.08 | **0.05** |
| parietal | theta | 949.221 | 19.313 | 6.113 | 3.16 | **0.005** |
| parietal | alpha | 107.475 | -5.777 | 5.434 | -1.06 | 0.3 |
| parietal | beta | 5.452 | -2.709 | 11.314 | -0.24 | 0.813 |
| parietal | gamma1 | 11.162 | -3.063 | 8.942 | -0.34 | 0.735 |
| parietal | gamma2 | 17.182 | 2.358 | 5.549 | 0.43 | 0.675 |
| temporal | delta | 303.915 | -8.097 | 4.918 | -1.65 | 0.115 |
| temporal | theta | 627.185 | 16.893 | 7.143 | 2.37 | **0.028** |
| temporal | alpha | 104.226 | -7.242 | 7.512 | -0.96 | 0.346 |
| temporal | beta | 1.939 | -1.313 | 9.984 | -0.13 | 0.897 |
| temporal | gamma1 | 0.016 | 0.113 | 9.37 | 0.01 | 0.991 |
| temporal | gamma2 | 2.558 | 0.73 | 4.834 | 0.15 | 0.881 |
| occipital | delta | 518.654 | -12.399 | 5.507 | -2.25 | **0.035** |
| occipital | theta | 647.114 | 20.908 | 8.314 | 2.51 | **0.02** |
| occipital | alpha | 96.845 | -7.833 | 8.051 | -0.97 | 0.342 |
| occipital | beta | 1.855 | 1.776 | 13.191 | 0.13 | 0.894 |
| occipital | gamma1 | 6.694 | -2.501 | 9.779 | -0.26 | 0.801 |
| occipital | gamma2 | 15.525 | 2.001 | 5.137 | 0.39 | 0.701 |

*Notes a: Type III SS = Type III Sum of Squares; b: estimate=regression estimate from the HLM model; c: SE=standard error of the estimate*

# Appendix, Part 10: Descriptive statistics for the predicted probability (%) of being classified as a responder, stratified by actual response group at study endpoint (non-responders, n = 20; responders, n = 7)

| Responder status | Predicted probability of being classified as a responder | | | |
| --- | --- | --- | --- | --- |
|  | gamma1 | | gamma2 | |
|  | median | interquartile range (Q1-Q3) | median | interquartile range (Q1-Q3) |
| non-responder | 7% | 5% - 27% | 4% | 2% - 14% |
| responder | 58% | 17% - 99% | 70% | 23% - 99% |

Note: Q1 and Q3 denote the 25^th^ and 75^th^ percentile values of the predicted probability distribution.
